# Cadmium in the shore crab *Carcinus maenas* along the Norwegian coast: geographical and seasonal variation and correlation to physiological parameters

**DOI:** 10.1007/s10661-018-6606-6

**Published:** 2018-03-27

**Authors:** Heidi Knutsen, Martin Wiech, Arne Duinker, Amund Maage

**Affiliations:** 1 National Institute of Nutrition and Seafood Research, 5002 Bergen, Norway; 2 University of Bergen, Allegt. 41, 2020 Bergen, Norway

**Keywords:** *Carcinus maenas*, Cadmium, *Cancer pagurus*, Shore crab soup, Seasonal variation, Physiological parameters

## Abstract

Previously, high concentrations of cadmium have been found in the hepatopancreas of the edible or brown crab (*Cancer pagurus*) sampled from positions north of about 67° N, compared to regions further south along the Norwegian coast, with no clear understanding why. In order to study a similar organism in the same ecosystem, the present study analyzed 210 shore crabs (*Carcinus maenas*) from four different locations along the Norwegian coast, two in the North and two in the South. The physiological variables size, sex, molting stage, hepatosomatic index, carapace color, and gonad maturation were registered, in attempt to explain the high inter-individual variation in cadmium levels in hepatopancreas. In contrast to the brown crabs, the shore crabs showed no clear geographical differences in cadmium concentrations. This indicates physiological differences between the two crab species. No clear and consistent correlations were found between cadmium levels and physiological parameters, except for sex, where cadmium concentration in hepatopancreas was twice as high in males compared to females. The cadmium levels also varied with season, with approximately 40 and 60% lower cadmium concentration in April than August for male and female shore crabs, respectively. None of the analyzed cadmium concentrations in muscle meat from claws exceeded EUs food safety limit, and low cadmium levels in soup prepared from shore crabs clearly indicated that this dish is not problematic regarding food safety.

**Electronic supplementary material:**

The online version of this article (10.1007/s10661-018-6606-6) contains supplementary material, which is available to authorized users.

## Introduction

It is well established that marine invertebrates such as crustaceans and mollusks can accumulate cadmium (Jennings and Rainbow 1979; Ray 1984; Wright 1976). A comparison of typical European foodstuffs revealed particularly high cadmium levels in crustaceans (EFSA 2012). As such, cadmium in crustaceans is of considerable interest both regarding toxic effects on the organisms itself (Weis 2012) as well as the suitability as food for humans. To ensure food safety, the European Commission has established upper limits for cadmium in several foods, where the limit for cadmium in claw meat of crustaceans is set at 0.5 mg Cd/kg wet weight (ww) (EU 2006). There is at present no legal limit for cadmium in the brown body meat, commonly consumed from crabs (Maulvault et al. 2013), although it mainly consists of hepatopancreas, where the majority of cadmium is accumulated (Bjerregaard 1990; Bjerregaard et al. 2005; Davies et al. 1981; Hutcheston 1974; Wiech et al. 2017) and gonad. The brown crab *Cancer pagurus* is commercially important, with an annual catch volume in Europe of approximately 50,000 t in total (Bakketeig et al. 2016) and rising to 10,000 t in Norway alone (Norwegian Directorate of Fisheries 2016). Findings of cadmium levels above the legal limit for claw meat in the brown crab from Northern Norway had a crucial negative impact on the crab fisheries in this area. To investigate the cadmium levels in brown crabs along the whole Norwegian coast, cadmium levels in brown meat and muscle meat from claws were measured of in a total of 475 frozen and cooked brown crabs sampled at 47 different sites along the Norwegian coast between July 2011 and January 2012. A pattern with significantly higher cadmium levels in brown crabs sampled from positions north of about 67° N (around Saltfjorden) was found compared to regions further south (Julshamn et al. 2012. The average cadmium concentrations in brown meat in frozen and cooked brown crabs sampled from positions north of Saltfjorden varied from 6.7 to 25 mg/kg wet weight and from 0.55 to 4.8 mg/kg wet weight in crabs sampled at positions south of Saltfjorden. In another survey, cadmium was measured in brown crabs sampled at 20 locations from Salten and further north to Vesterålen (Frantzen et al. 2015). In agreement with the survey from 2011 and 2012, high levels of cadmium were found varying from 2.4 to 17 mg/kg wet weight in brown meat. No difference was seen between samples from inner fjord and outer coast localities.

Several follow-up studies have been performed in attempt to explain the elevated cadmium levels in brown crabs from Northern Norway compared to the rest of the Norwegian coast. However, no obvious point source from industry has been determined responsible for the high cadmium levels found in the brown crabs (Falk 2012). Further, measurements of cadmium in surface water, groundwater, soil, and bedrock have not displayed elevated cadmium levels in the Salten region (Finne 2013). Surveys have shown relatively low cadmium levels in fish species and blue mussels from Northern Norway (Julshamn et al. 2013a; Ørnsrud and Måge 2012; Foldøy Tverdal 2012), and no correlation to the elevated values in the brown crabs were found.

As reported for the brown crab (Wiech et al. 2017), also the shore crab *Carcinus maenas* is able to accumulate high levels of cadmium in heptaopancreas (Rainbow et al. 1990). As these species are also sharing parts of the same ecological niche, a comparison of their cadmium levels is of considerable interest. The smaller shore crab is found subtidally as well as intertidally on all shores (Crothers 1968), while the brown crab is abundant from the shallow sublittoral to depths of about 100 m (Neal and Wilson 2008). Shore crab is considered a delicacy in Spain and Portugal, with commercial fisheries yield of up to 900 t per year for France, Portugal, and Spain together (Klassen and Locke 2007). The culinary popularity is also increasing in Norway, especially as a base for shore crab soup. In terms of food safety, it is therefore important to study the cadmium levels in shore crabs. Comparison of brown and shore crabs geographical cadmium pattern would contribute to explaining the high cadmium levels in brown crabs north of 67° N.

In brown crabs, concluding studies are hampered by large inter-individual variability in cadmium between brown crabs from the same geographical areas, with especially high variation in the hepatopancreas (Davies et al. 1981; Maulvault et al. 2012; Wiech et al. 2017). Shore crabs have also shown to display large variability in their cadmium levels (Bjerregaard 1982, 1990, 1991; Bjerregaard and Depledge 2002; Bondgaard et al. 2000; Nørum et al. 2003). The high variation could be caused by biological factors. Laboratory studies have shown relationships between cadmium levels and physiological variables such as sex (Bjerregaard et al. 2005), size (Bjerregaard and Depledge 2002), molt stage (Bondgaard et al. 2000; Bondgaard and Bjerregaard 2005; Nørum et al. 2005), ovarian maturation (Bondgaard et al. 2000), and variables indicating the condition of the crab (Bjerregaard 1991), like water content in tissues (Bjerregaard and Depledge 2002). As observed by Bjerregaard et al. (2005), the tissue cadmium content may also vary seasonally. Cadmium bioaccumulation increases with increasing temperature (Ray 1984), and reduced salinity stimulate uptake of anionic cadmium species in brachyuran crabs (Wright 1977; Burke et al. 2003).

In the present study, cadmium levels in shore crabs were investigated for comparison to the problematic high levels in brown crabs, which have many parallels in physiology as well as a similar ecological niche as the shore crab. Also, the cadmium levels in shore crabs were investigated due to food safety reasons. This paper describes geographical (investigation 1) and seasonal (investigation 2) variations in cadmium levels in shore crabs sampled from four different sites along the Norwegian coast. In addition, the study examined effects of different physiological parameters on individual cadmium levels (investigation 3). Lastly, cadmium concentrations in shore crab soup (investigation 4) are described for an evaluation of food safety.

## Materials and methods

### Sampling of biological material

**Fig. 1:**
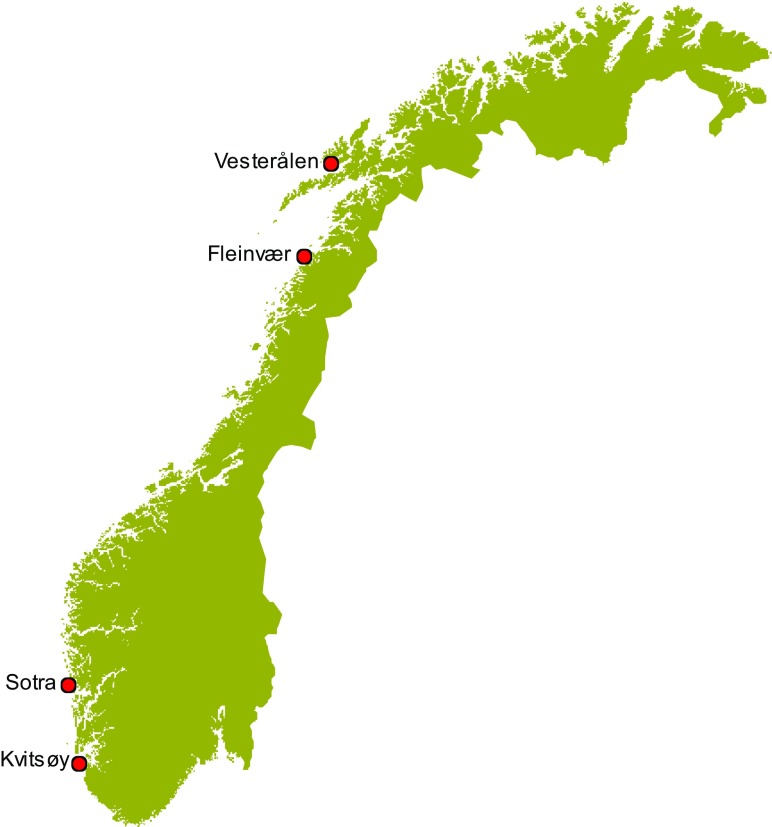
The Norwegian coastline showing the four sampling areas Kvitsøy, Sotra, Fleinvær, and Vesterålen

Male and female shore crabs *Carcinus maenas* with carapace width (CW) varying from 29 to 88 mm were caught along the Norwegian coast in baited pots at approximately 1–5 m water depth between March and August 2016. The sampling locations (Fig. 1) were chosen according to earlier studies on cadmium in the brown crab (Julshamn et al. 2012; Wiech et al. 2017).

### Investigation 1: geographical variation in cadmium

Shore crabs of similar size (CW 62 ± 7 mm (mean ± SD)) were collected from Kvitsøy (59° N), Sotra (60° N), Fleinvær (67° N), and Vesterålen (68° N) during the spring of 2016 (March–May). From each site, 30 shore crabs were collected with equal sex distribution, except from Fleinvær, with 27 male and three female crabs. Although an effort was made to keep experimental groups as uniform as possible, there was some variation in sizes of specimens, with the male shore crabs from Fleinvær being significantly larger (70 ± 3 mm) than the other males (64 ± 4 mm), and the females from Kvitsøy being significantly smaller (50 ± 3 mm) than the other females (58 ± 4 mm).

### Investigation 2: seasonal variation in cadmium

To investigate whether the cadmium content in shore crabs varies with season, 30 shore crabs (61 ± 5 mm) were collected in the end of August 2016 (Sotra-August) in addition to the 30 shore crabs (61 ± 5 mm) collected earlier from Sotra in the middle of April 2016 (Sotra-April). The sex distribution was equal in both groups.

### Investigation 3: physiological variables and their effect on cadmium

To examine the potential effect of size on cadmium levels, in addition to the 30 crabs from investigation 1, 30 shore crabs were collected from Sotra and Vesterålen during April–May 2016 to obtain the largest size range possible (CW from 29 to 88 mm and whole body weight from 6 to 170 g).

To investigate the correlation of cadmium levels to further physiological variables, several individual physiological variables were measured in all of the sampled shore crabs:

After arrival at the National Institute of Nutrition and Seafood Research (NIFES), the crabs were sacrificed following the guidelines in WHO/FAO (2012), by piercing the nerve ganglia as described by Baker (1955) and dissected freshly, as freezing and boiling may affect cadmium levels in crab tissues (Wiech et al. 2017). The same observer recorded all visually examined measures to minimize bias.

For each individual, carapace width, whole body wet weight, sex, damage on the exoskeleton, and missing legs and/or claws were recorded. One of the following carapace colors was assigned: green, brown, blue/black, orange, or red. For female shore crabs, the presence of sperm plug was noted.

Crabs were assigned to one of four different molting stages (early post molt, recent molt, inter-molt, or degraded) by examination of carapace hardness, levels of biofouling, and visual indices according to Haig et al. (2016).

To determine the hepatosomatic index (HSI), an indication of lipid stores, the hepatopancreas was removed and weighed, and HSI was calculated:

1 $$ \mathrm{HSI}=\frac{m_{\mathrm{HP}}}{m_{\mathrm{whole}\ \mathrm{body}}-{m}_{\mathrm{HP}}}\times 100\% $$

Where *m*_HP_ and *m*_whole body_ are individual hepatopancreas and whole body wet weights, respectively. Hepatopancreas samples were individually homogenized and kept for analysis.

Gonads were staged as described in Haig et al. (2016) and removed from mature female crabs and pooled for analysis for each location separately. Pooled samples were also prepared for muscle meat for each sampling area and sex. After the wet weight was obtained for all samples, they were frozen and subsequently freeze-dried (Freezone 18 l by Labconco, Kansas, USA) to determine the dry weight content. The water content (WW%) was obtained for all samples:

2 $$ \mathrm{WW}\%=100\%-\frac{\mathrm{wet}\ \mathrm{weight}}{\mathrm{dry}\ \mathrm{weight}}\times 100\% $$

Where wet weigh*t* and dry weight are sample weight before and after freeze drying, respectively.

### Investigation 4: cadmium in shore crab soup

To measure possible cadmium exposure from shore crab soup, triplicates of soups were made using crabs from Sotra and Vesterålen, separately. From Sotra, 30 shore crabs were collected, with equal sex distribution (CW = 62 ± 6 and 58 ± 4 mm for male and female crabs, respectively). From Vesterålen, the selection was limited to 15 male crabs (CW = 74 ± 2 mm). Approximately, the same weight of shore crabs were used in each triplicate (397 ± 13 and 457 ± 1.0 g for Vesterålen and Sotra, respectively).

After the crabs were sacrificed, they were cut in half and fried while crushing with a solid kitchen spoon in a saucepan with heated vegetable oil with no salt. After about 5 min, the crabs turned red, and water was added to cover the crabs (4–5 dl). After 30 min of boiling, the soup was sifted off and cooled for freeze-drying and homogenization.

### Chemical analysis

Freeze-dried tissue and soup samples were homogenized and prepared for metal analysis using ICP-MS (iCAP Q) as described by Julshamn et al. (2007). The method was accredited according to NS-EN 17025, and the quality of the metal measurements was assured by the use of the certified reference materials (CRM), Tort-3 (Lobster Hepatopancreas, National Research Council, Canada) and 1566b-O.T. (Oyster Tissue, National Institute of Standards and Technology, Gaithersburg, USA). Average values for all metals were within 20% of the certified values, and the dry weight (dw)-based quantification limit (LOQ_dw_) for cadmium was set to 0.005 mg/kg with standard sample size (0.2 g). All individual samples were over the wet weight-based quantification limit (LOQ_ww_), calculated as follows:

3 $$ {\mathrm{LOQ}}_{ww}={\mathrm{LOQ}}_{\mathrm{dw}}\times \frac{\mathrm{wet}\ {\mathrm{weight}}_{\mathrm{sample}}}{dry\ {weight}_{sample}} $$

### Statistical analysis

When necessary, the data was box-cox transformed to obtain normality and homogeneity of variances, tested for by normal plots and Levene’s *F* test, respectively. Results were evaluated using analysis of variance (ANOVA) followed by Tukey HSD post hoc test as the multiple comparison procedure. The significance level was 0.05. Simple linear regression analysis was performed by using Pearson’s linear correlation (STATISTICA v. 13.1, ©1984–2016 by Statsoft, Tulsa, USA).

## Results

The mean cadmium concentrations in hepatopancreas was 1.1 ± 1.2 mg/kg ww (mean ± SD) corresponding to 3.4 ± 4.1 mg/kg dw (mean ± SD) and ranged from 0.046 to 11 mg/kg ww corresponding to 0.13 to 39 mg/kg dw, underlining the high individual variability. Furthermore, the cadmium concentrations were higher for male than female shore crabs for all locations with 1.3 ± 1.3 mg/kg ww (mean ± SD) corresponding to 4.3 ± 4.6 mg/kg dw (mean ± SD) and 0.61 ± 0.79 mg/kg ww (mean ± SD) corresponding to 2.0 ± 2.5 mg/kg dw (mean ± SD) for male and female shore crabs, respectively (Table 1). Cadmium concentrations in muscle meat and gonads were significantly lower than in hepatopancreas for both sexes (*p* < 0.0001) with 0.0027 ± 0.0017 mg/kg ww (mean ± SD) corresponding to 0.0112 ± 0.0069 mg/kg dw (mean ± SD) for muscle meat in claws, and 0.0149 ± 0.0055 mg/kg ww (mean ± SD) corresponding to 0.036 ± 0.014 mg/kg dw (mean ± SD) for gonads, respectively (supplementary Tables 1 and 2). In muscle meat, there was no significant difference between males and females. For the analyzed tissues, the cadmium distribution was 99.7% in hepatopancreas and 0.3% in muscle meat from claws for the male crabs. For the female shore crabs, the cadmium distribution was 92% in hepatopancreas, 0.1% in muscle meat from claws, and 7.9% in gonads. The water content in these tissues was 32 ± 5.3 and 24 ± 2.1% for hepatopancreas and muscle meat for male shore crabs; and 31 ± 4.3, 24 ± 1.4, and 42 ± 5.4% for for hepatopancreas, muscle, and gonads for the female shore crabs. More detailed values on weight, carapace width, dry matter in hepatopancreas, and hepatosomatic index are presented in Table 2.

**Table 1:** Cadmium concentrations (mg/kg) based on wet weight (ww) and dry weight (dw) in hepatopancreas of shore crabs (*Carcinus maenas*) from the Norwegian coast

Area	Male					Female				
	*N*	Mean ± SD (ww)	Range (ww)	Mean ± SD (dw)	Range (dw)	*N*	Mean ± SD (ww)	Range (ww)	Mean ± SD (dw)	Range (dw)
Kvitsøy	15	0.98 ± 0.68	0.11–2.7	3.0 ± 2.1	0.38–7.6	15	0.45 ± 0.57	0.056–2.3	1.4 ± 1.7	0.16–6.6
Sotra-April	33	1.0 ± 0.59	0.14–2.4	3.3 ± 2.0	0.36–7.4	27	0.91 ± 1.1	0.059–4.0	2.8 ± 3.2	0.19–12
Fleinvær	27	2.4 ± 2.2	0.37–11	7.7 ± 7.7	1.3–39	3	0.58 ± 0.35	0.20–0.87	2.0 ± 1.0	0.90–2.7
Vesterålen	42	1.1 ± 1.0	0.16–4.1	3.9 ± 3.6	0.37–14.9	18	0.47 ± 0.54	0.048–2.4	1.6 ± 1.7	0.13–7.5
Sotra-August	15	0.90 ± 0.78	0.093–3.4	3.2 ± 3.0	0.44–12.4	15	0.39 ± 0.60	0.046–2.3	1.5 ± 2.3	0.18–9.1
All areas	132	1.3 ± 1.3	0.093–11	4.3 ± 4.6	0.36–39	78	0.61 ± 0.79	0.046–4.0	2.0 ± 2.5	0.13–12

Mean ± standard deviation (SD) and concentration ranges are given for each group

**Table 2:** Weight (g), carapace width (CW mm), dry matter content in hepatopancreas (DM, %), and hepatosomatic index (HSI, %) of shore crabs (*Carcinus maenas*) from different sites along the Norwegian coast

Area	Male				Female			
	Weight (g)	CW (mm)	DM (%)	HSI (%)	Weight (g)	CW (mm)	DM (%)	HSI (%)
Kvitsøy	58 ± 11	6.2 ± 0.35	32 ± 5.1	5.2 ± 1.2	26 ± 4.5	5.0 ± 0.34	33 ± 5.5	5.4 ± 0.99
Sotra-April	73 ± 26	6.7 ± 0.96	31 ± 5.5	6.1 ± 1.3	30 ± 12	5.2 ± 0.79	31 ± 4.3	7.9 ± 2.4
Fleinvær	86 ± 14	7.0 ± 0.33	31 ± 3.9	7.0 ± 1.2	59 ± 17	6.4 ± 0.56	29 ± 8.3	8.0 ± 0.89
Vesterålen	74 ± 44	6.4 ± 1.4	32 ± 6.7	7.8 ± 2.5	39 ± 11	5.6 ± 0.52	31 ± 5.8	7.7 ± 1.6
Sotra-August	54 ± 15	6.3 ± 0.56	28 ± 4.9	7.5 ± 1.4	42 ± 6.5	5.8 ± 0.32	27 ± 4.3	9.4 ± 1.0
All areas	72 ± 31	6.6 ± 1.0	31 ± 5.6	6.9 ± 2.0	35 ± 12	5.4 ± 0.67	31 ± 5.4	7.7 ± 2.1

Mean ± standard deviation (SD) and concentration ranges are given for each group

### Investigation 1: geographical variation in cadmium

**Fig. 2:**
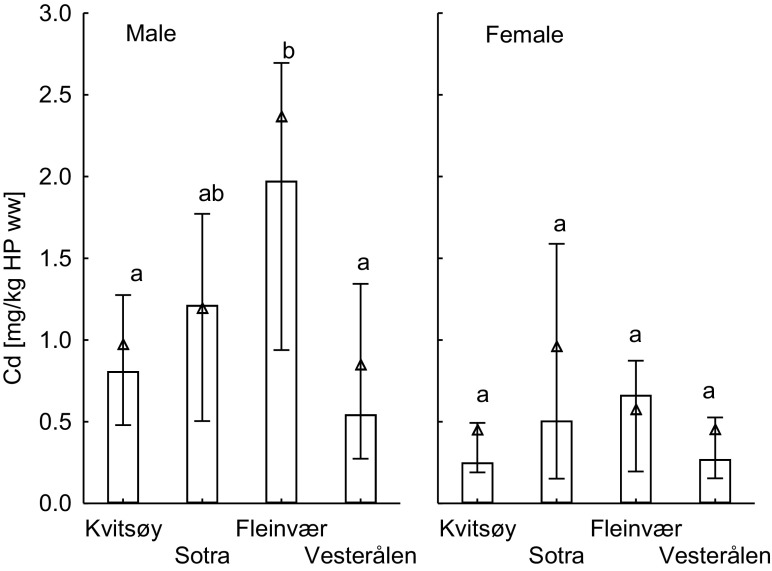
Geographical variation in cadmium concentrations in hepatopancreas (mg/kg wet weight) of male (left panel, *n* = 72) and female (right panel, *n* = 48) crabs collected from Kvitsøy and Sotra in south, and Fleinvær and Vesterålen in north (median ± 25% percentile is given, and triangle symbols shows the mean for each location; sampling points with no letters in common show statistically significant differences)

Cadmium concentrations in hepatopancreas based on dry weight and wet weight (Table 1) did not vary significantly between the different sampling areas for the female shore crabs (Fig. 2). Male crabs from Fleinvær however, had significantly higher wet weight-based cadmium concentrations in hepatopancreas compared to the male crabs from Kvitsøy in Southern Norway (*p* < 0.01), and Vesterålen in Northern Norway (*p* < 0.001). On dry weight basis, cadmium concentrations found in male crabs from Fleinvær were in addition higher than in male crabs from Sotra (*p* < 0.02). The male crabs from Vesterålen did not have higher cadmium levels compared to the crabs sampled further south (Fig. 2). There was no significant geographical difference in cadmium levels in muscle and gonads for neither males nor females (*p* > 0.05) (supplementary Tables 1 and 2).

### Investigation 2: seasonal variation in cadmium

**Fig. 3:**
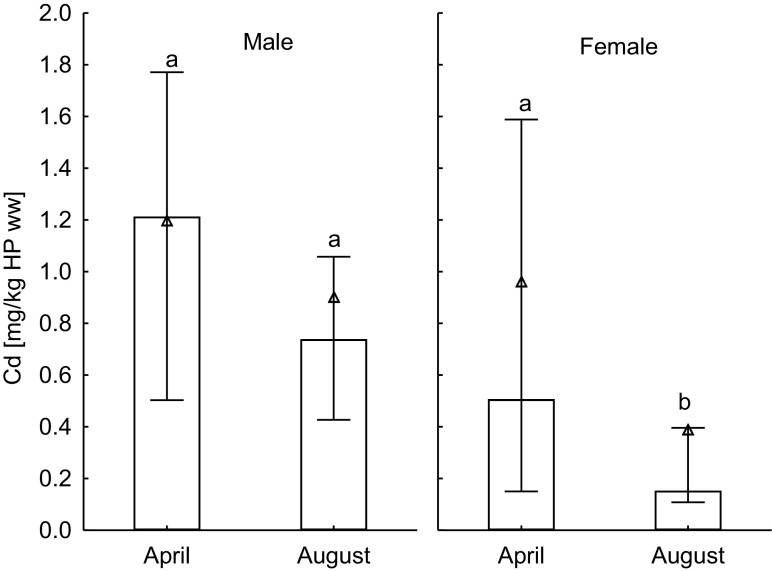
Cadmium concentrations in hepatopancreas (mg/kg wet weight) of male (left panel, *n* = 30) and female (right panel, *n* = 30) crabs collected from Sotra in April and August (median ± 25% percentile is given, and triangle symbols shows the mean for each location; sampling points with no letters in common show statistically significant differences)

Crabs sampled in August had lower wet- and dry weight-based cadmium concentrations in hepatopancreas than in April, and the difference was significant for the female crabs (*p* < 0.03), while not significant for the male crabs (*p* > 0.05) (Fig. 3). The total cadmium content in hepatopancreas was not statistically significantly different between August and April (*p* > 0.1). However, there was a clear trend in measured cadmium concentration, with about two times lower concentrations for both sexes in August than in April. Statistically, the difference was probably covered by the large variation between individuals. There was no significant seasonal variation in cadmium levels in muscle and gonads (*p* > 0.7).

### Investigation 3: physiological variables and their effect on cadmium

The size parameters carapace width and whole body weight were strongly correlated for both sexes (*r*^2^ = 0.90, *p* < 0.001 and *r*^2^ = 0.88, *p* < 0.001 for male and female shore crabs, respectively (Supplementary Table 3). Carapace width was chosen as the main size parameter for further examination, and it was positively correlated with water content in hepatopancreas for both male (*r*^2^ = 0.25, *p* < 0.0001) and female (*r*^2^ = 0.32, *p* < 0.0001) shore crabs. The hepatosomatic index was negatively correlated with carapace width for the males (*r*^2^ = − 0.35, *p* < 0.0001), though not for females (*r*^2^ = 0.026, *p* < 0.2).

**Fig. 4:**
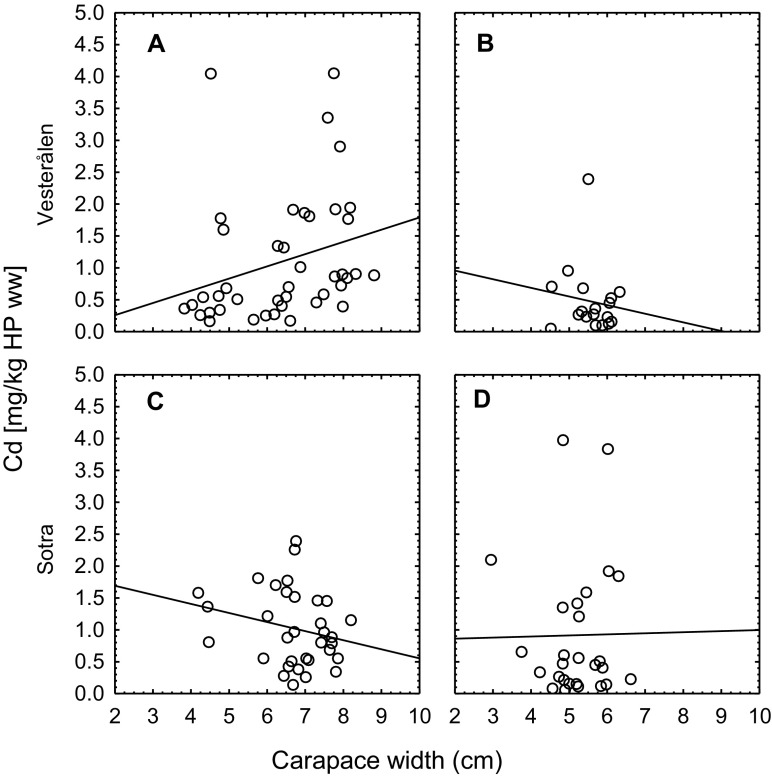
Relationship between cadmium concentration in hepatopancreas (mg/kg wet weight) and carapace width of male (*n* = 75) (**a**, **c**) and female (*n* = 45) (**b**, **d**) shore crabs from Vesterålen in north (upper panel) and Sotra in south (lower panel)

Overall, size was not correlated with cadmium concentrations in hepatopancreas despite the relatively large size variation for the shore crabs from Sotra and Vesterålen (Fig. 4)—a difference of about three times between maximum and minimum carapace width. For the male crabs from Vesterålen, there was a weak significantly positive correlation between carapace width and cadmium concentrations (*r*^2^ = 0.15, *p* < 0.01 on wet weight basis and *r*^2^ = 0.29, *p* < 0.001 on dry weight basis). The correlation was pronounced for carapace width and total cadmium content in hepatopancreas (*r*^2^ = 0.47, *p* < 0.0001). For male crabs from Sotra, cadmium concentrations were not significantly correlated with carapace width, but the total cadmium content in hepatopancreas showed a weak correlation with carapace width (*r*^2^ = 0.13, *p* < 0.05). The cadmium concentrations were not significantly correlated with carapace width for female crabs from neither Vesterålen nor Sotra. However, the cadmium content was weakly correlated with carapace width for the females from Sotra (*r*^2^ = 0.15, *p* < 0.05).

Overall, there was no clear correlation between cadmium concentration in hepatopancreas (mg/kg ww) and the physiological variable water content. Only for male shore crabs from Vesterålen a weak significantly positive correlation (*r*^2^ = 0.20, *p* < 0.05) was observed. On dry weight basis (mg Cd/kg w), the correlation was pronounced (*r*^2^ = 0.39, *p* < 0.001). In addition, there was a significantly positive correlation between water- and cadmium content in hepatopancreas for the male shore crabs from Vesterålen (*r*^2^ = 0.30, *p* < 0.001).

Male shore crabs had higher cadmium concentrations and a higher total amount of cadmium in hepatopancreas in all experimental groups (*p* < 0.003). Physiological variables that were significantly different between male and female shore crabs might be of importance for the different cadmium levels. The male crabs were significantly larger than females in both CW and body weight (*p* < 0.0001), and the females had a significantly lower hepatosomatic index (*p* < 0.02) (Table 2). However, there was no clear correlation between HSI and cadmium levels when sexes were segregated, except for a significant negative correlation between cadmium concentration on dry weight basis and HSI for the male shore crabs from Vesterålen: *r*^2^ = 0.27, *p* < 0.001). However, a relationship between cadmium concentration and HSI was indicated, as the females had significantly higher HSI and lower cadmium levels in hepatopancreas. Additionally, both sexes had significantly higher HSI (*p* < 0.0003) and lower cadmium concentration in August than April at Sotra. For the females from Sotra-April and Sotra-August in total, the correlation between cadmium concentration and HSI was significantly negative (*r*^2^ = 0.24, *p* < 0.006), whereas there was no significant correlation between total cadmium content in hepatopancreas and HSI (*p* > 0.05). Female crabs showed a seasonal trend in dry matter content in Sotra with a lower dry matter content in August (*p* = 0.066), while this was not observed in male crabs. In male crabs, the dry matter content increased significantly from recent molt to inter-molt crabs, while this was only visible as a trend in females (supplementary Fig. 1).

The proportion of red females was larger than for males (approximately 65 and 50%, respectively), but no significant correlation was found between carapace color and cadmium levels (*p* > 0.05) (supplementary Fig. 2). There were no clear and consistent relationships between cadmium and other physiological variables such as molt stage (supplementary Fig. 3), gonad maturation stage (supplementary Figs. 4 and 5), and the presence of sperm plugs, probably linked to high variation in Cd values and low variation in the measurement of the parameters.

### Investigation 4: cadmium in shore crab soup

The cadmium concentrations in shore crab soups from Sotra and Vesterålen ranged from 17 to 79 μg/kg ww, with average values of 28 ± 11 and 63 ± 14 μg/kg ww, respectively. In total, the average cadmium concentration was 44 ± 22 μg/kg ww soup. Based on cadmium levels in hepatopancreas in shore crabs from Sotra and Vesterålen (Table 1), it was calculated that approximately 62% of the crabs’ cadmium levels were extracted into the soup during the cooking process.

## Discussion

### Geographical variation in cadmium

The main objective in the present study was to investigate whether the shore crab shows as large differences in cadmium concentrations in hepatopancreas as have been found earlier in brown crabs from northern and southern sites along the Norwegian coast. The cadmium concentrations in the shore crabs in this study did not follow such a clear geographical pattern. Only males from one of the two locations in the North showed significantly higher concentrations. This is in contrast to the brown crab, where concentrations in the brown meat were significantly higher in Northern Norway, compared to Southern Norway (Julshamn et al. 2012). Except for the relatively high cadmium concentrations in male shore crabs from Fleinvær, the cadmium levels in the analyzed tissues in the present study correspond fairly well to earlier published values for shore crabs from Denmark and Scotland (Depledge and Bjerregarrd 2002; Bjerregaard 1982, 1990; Rainbow 1985). The male shore crabs from Fleinvær were larger than the other males, and one reason for their high cadmium levels may be that they have foraged on different and potentially larger and older prey, with potentially higher cadmium levels. It is also possible that the cadmium levels are higher in this area, although the cadmium concentrations in the females from Fleinvær were not correspondingly high. However, this may be due to the low sampling size of female shore crabs from this area, with only three females from Fleinvær. Brown crabs from the same area did not show elevated cadmium levels compared to other samples in the North (Julshamn et al. 2013b; Julshamn et al. 2012).

The differences in geographical cadmium pattern between shore crabs and brown crabs from the Norwegian coast may be explained by several causes. As food presumably is the most important cadmium source for crabs (Davies et al. 1981; Bjerregaard et al. 2005), differences in diet might be of importance for the different cadmium levels between shore crabs and brown crabs sampled along the Norwegian coast. Compared to the cadmium levels in the brown crab (Julshamn et al. 2012), the levels in the shore crab in this study were low along the whole Norwegian coastline. The brown crab generally consumes larger organisms (Mascar and Seed 2001) with potentially higher cadmium levels. Further, differences in diet might be a result of their different distribution in the water column, as the shore crab generally lives in shallower waters (Crothers 1968; Neal and Wilson 2008), where different prey species might be abundant.

Crabs also accumulate cadmium from the water phase (Jennings and Rainbow 1979; Weis 2012; Davies et al. 1981), and it is possible that differences in cadmium accumulation from the water is associated with the higher cadmium levels in brown crabs compared to shore crabs. As the brown crab is more abundant in deeper water, it is possible that it is more exposed to the cadmium rich deep-water (Falk and Nøst 2013, Janssen et al. 2014) than the shore crab. However, the cadmium levels in brown meat in brown crabs from Northern Norway are shown to be higher in males (Frantzen et al. 2015), even though females generally migrates to a greater extent to deeper waters where the cadmium concentrations potentially are higher (Ungfors et al. 2007; Falk and Nøst 2013). Further, several studies indicate that the rates of cadmium accumulation from the water phase to the hepatopancreas are too low to explain the high cadmium levels in hepatopancreas and/or brown meat (Bjerregaard et al. 2005; Davies et al. 1981; Jennings and Rainbow 1979; Nørum et al. 2005).

It might be an explanatory feature that the shore crab could probably be better adapted to the cold climate in Northern Norway compared to the brown crab. The water temperature along the Norwegian coast generally decreases with increasing latitude, and the mean temperature for 2015 to 2017 at Sognesjøen (61° N), a station close to our sampling sites in Southern Norway, was 10.0 °C, ranging from 5.8° to 15.6 °C at a depth of 5 m (IMR 2018). At a station in the proximity of our northernmost site, Eggum (68° N), a mean temperature of 8.3 °C with, ranging from 4.7° to 12.3 °C was measured for 2015 to 2017 at a depth of 5 m (IMR 2018). The shore crab is known to be very robust and survives a wide range of temperatures from approximately 0 to 35 °C and tolerates salinities from 4 to 52 ‰ (Klassen and Locke 2007). This suggests that the shore crab grows equally good in northern and Southern Norway. There is evidence that brown crabs do not feed at all at temperatures below 5 °C, as well as migration is limited (Karlsson and Christiansen 1996). Further, a survey of brown crabs has shown that they molt less frequently in northern compared to Southern Norway (Snorre Bakke, personal communication, January 23, 2017). Consequently, the growth rate will be relatively lower for brown crabs from Northern Norway. As such, a brown crab of a given size from Northern Norway might have had longer time to accumulate metals such as cadmium and will consequently have higher cadmium levels compared to a brown crab of similar size sampled further south. This will probably not apply for the shore crab, under the assumption that shore crabs have similar growth rates in the north and south. Further, Bergey and Weis (2007) have suggested molting as a mechanism for depuration of lead for the fiddler crab *Uca pugnax.* If molting is a feasible mechanism for crabs to also departure cadmium, it is possible that lower molting frequency for brown crabs from Northern Norway results in less cadmium excretion and thereby higher cadmium concentrations compared to brown crabs from Southern Norway.

### Seasonal variation in cadmium

In agreement with findings from Bjerregaard et al. (2005), the results in the present study showed that the cadmium concentrations in hepatopancreas vary with season. The concentration was significantly lower for female shore crabs sampled in August than April. The seasonal variation might be explained in terms of changing bioavailability of the metal due to changing physicochemical conditions of the environment as well as changing physiological state of the individuals. With rising water temperature, the crabs activity will probably increase (Klassen and Locke 2007; Griffen et al. 2012), which may lead to increased food intake. This is indicated by significantly higher hepatosomatic index for both sexes in August than April. With higher HSI, the lipid stores consequently increase, which seems to lower the cadmium concentration by dilution, as indicated by the significantly negative correlation between HSI and cadmium concentration for the females sampled in August and April in total. Further, the higher concentrations of cadmium in hepatopancreas in April could be explained by the tendency to higher water content in hepatopancreas in the crabs sampled in August. This is consistent with the total content of cadmium not differing between the 2 months. In addition, the lower cadmium levels in August might be a consequence of a potentially shorter biological half-life of cadmium as the temperature increases during summer, with subsequently higher activity among the crabs, as discussed by Bjerregaard et al. (2005).

Seasonal changes in physiological variables such as ovarian maturation and molt stage (Bondgaard et al. 2000: Bondgaard and Bjerregaard 2005; Nørum et al. 2005) might also influence the cadmium levels, but the span in variation for these parameters was too low to reveal any effects. In agreement with the results in the present study, elevated cadmium levels are reported for the American oyster (*Crassostrea virginica*) in April with a decline throughout the summer (Frazier 1979).

As the bioavailability of cadmium increase with temperature (Klassen and Locke 2007; Ray 1984; Rainbow 1997; Burke et al. 2003), the cadmium accumulation from the water phase would probably be higher for the shore crabs in August than April. However, the cadmium levels were not elevated in August for the shore crabs in this study. Therefore, cadmium uptake from the water phase does not explain the observed variations.

### Physiological variables and their effect on cadmium

For both sexes, the size parameters were positively correlated with each other and with water content in hepatopancreas, in agreement with other studies (Bjerregaard and Depledge 2002; Nissen et al. 2005). For the male shore crabs, the HSI was negatively correlated with size, which indicates that the relative energy reserves decrease with increasing size. In concordance to Nørum et al. (2003), we found a trend towards a higher dry matter content in hepatopancreas in inter-molt crabs in comparison to recent molt crabs. This is most likely a result of active foraging.

We found an indication of cadmium accumulation over the crab’s lifetime, as the total amount of cadmium was correlated with size. However there was no consistent effect of size on cadmium concentrations even though the range in size was relatively large. The increase of water content with size might mask the increase of the wet weight-based concentration. Little or no correlation between size and cadmium concentrations has been found for shore crabs from Denmark (Bjerregaard and Depledge 2002; Nissen et al. 2005), king crabs (*Pseudocarcinus gigas)* from Australia (Turoczy et al. 2001), and brown crabs from Norway (Julshamn et al. 2012). It is possible that crabs excrete cadmium during molting (Bergey and Weis 2007), which could explain why the cadmium concentrations do not increase considerably with size. However, this needs to be elucidated further.

There was a clear difference between the sexes regarding cadmium concentrations in hepatopancreas. The cadmium concentration was more than twice as high for the male shore crabs. In correspondence with these results, Bjerregaard et al. (2005) also found generally lower cadmium concentration in female hepatpancreas, compared to male shore crabs. As food presumably is the most important cadmium source for shore crabs (Bjerregaard et al. 2005; Pedersen et al. 2014), it is possible that differences in foraging strategy between male and female shore crabs may lead to differences in accumulation of cadmium. The sexes behave differently in the coastal zone, where the females generally stay at deeper waters than the males (Reid et al. 1997). Furthermore, the males are generally larger, with bigger and presumably stronger claws, which enables foraging on larger organisms (Kaiser et al. 1990) with potentially higher cadmium levels.

The cadmium concentration in muscle meat from claws was not significantly different between male and female shore crabs. The majority of the accumulated cadmium was measured in hepatopancreas, and the whole body cadmium distribution was approximately 92, 7.9, and 0.10% in hepatopancreas, female gonads, and muscle meat from claws, respectively, for the analyzed tissues. The cadmium distribution in the analyzed tissues was similar to other studies on both shore crabs and brown crabs (Bondgaard and Bjerregaard 2005; Bjerregaard et al. 2005; Weis 2012; Frantzen et al. 2015; Wiech et al. 2017).

Except for a clear relationship between cadmium concentrations and sex and weak correlations between hepatosomatic index and carapace width and cadmium, there was no correlation between cadmium concentrations and the registered physiological variables. Limited range in visually assessed parameters such as carapace color, number of legs and claws, molting stage, presence of sperm plugs, and gonad maturation might be the reason why no relationships between cadmium levels and these individual parameters were found. Other studies have shown higher cadmium accumulation rates for crabs in early post-molt and early ovarian maturation stages when exposed to cadmium in water (Bondgaard et al. 2000; Bondgaard and Bjerregaard 2005; Nørum et al. 2005), and green shore crabs seems to accumulate more cadmium than red shore crabs (Nissen et al. 2005; Styrishave et al. 2000).

### Cadmium in shore crab soup

Even though the amount of cadmium extracted from the crabs to the soup was relatively high, the cadmium concentrations in the prepared shore crab soup were low. Therefore, shore crab soup was considered to be safe regarding cadmium exposure. Based on the highest measured cadmium concentration of 79 μg Cd/kg ww, a portion size of 100 g would constitute approximately 5% of the tolerable weekly intake (TWI) for a person weighing 70 kg, based on the TWI of 2.5 μg Cd/kg body weight (EFSA 2009), set by the European Food Safety Authority (EFSA). However, it is estimated that the average cadmium exposure from food is approximately 1.7 μg/kg body weight per week for an adult Norwegian person (VKM 2015). Taking this into consideration, the additional dietary cadmium exposure allocated to other dietary sources is 56 μg per week given a body weight of 70 kg, which corresponds to approximately seven portions of shore crab soup per week. As such, shore crab soup is not considered problematic regarding food safety. Furthermore, a cooking time of 30 min may be excessive as the soup may become bitter during the long cooking process (personal observation). However, it was chosen as worst-case scenario to ensure sufficient cadmium extraction. In addition, all the pooled samples were under the legal limit of 0.5 mg Cd/kg ww in claw meat for humane consume, set by EU (EU 2006).

## Conclusion

The cadmium concentrations in shore crabs in this study were very low in muscle meat from claws and between 0.046 mg Cd/kg ww and 11 mg/kg ww in hepatopancreas. There was no clear geographical difference with latitude as opposed to earlier findings in the brown crab. Possible explanations for this may be that these species have different feeding habits or that the shore crab is better adapted to the colder climate in Northern Norway. Sex had a clear impact on the cadmium levels in hepatopancreas, as the male shore crabs had approximately twice as high cadmium levels compared to the females. No clear and consistent correlations were found between cadmium and other registered individual variables, but some minor relationships were seen with an indication of cadmium accumulation over time as well as a weak relationship between cadmium concentrations and fluctuating water contents of tissues. Cadmium concentrations were lower in August than in April. Most of the total amount of cadmium was allocated in the hepatopancreas while muscle meat and gonads of females contributed together with less than 10%. None of the measured cadmium levels exceeded EUs legal limit of 0.5 mg Cd/kg ww set for claw meat for human consumption.

Low cadmium levels in shore crab soup make it a safe food item regarding cadmium and food safety.

## Electronic supplementary material

ESM 1 (DOCX 176 kb)
