# Supplementary material for: Cadmium in the shore crab Carcinus maenas along the Norwegian coast: geographical and seasonal variation and correlation to physiological parameters
Source: Environ Monit Assess. 2018 Mar 27;190(4):253. doi: 10.1007/s10661-018-6606-6 (PMC5871639; doi:10.1007/s10661-018-6606-6)
Supplement: Supplementary file 1 — (DOCX 176 kb) [file 10661_2018_6606_MOESM1_ESM.docx]

**Cadmium in the shore crab *Carcinus maenas* along the Norwegian Coast: geographical and seasonal variation and correlation to physiological parameters**

**Heidi Knutsen^1^, Martin Wiech^1^, Arne Duinker^1^ and Amund Maage^1, 2^**

^1^National Institute of Nutrition and Seafood Research, 5002 Bergen, Norway

^2^University of Bergen, Allegt. 41, 2020 Bergen

**Corresponding author:** Heidi Knutsen, e-mail: [hknutsen10@gmail.com](mailto:hknutsen10@gmail.com)

**Journal:** Environmental Monitoring and Assessment

**Supplementary Material**

Supplementary Fig. 1 Dry matter content of hepatopancreas of male and female shore crabs at different moulting stages. Bars denote the mean concentration and whiskers one standard deviation. The number of crabs within each category is given. Different letters indicate significant differences

Supplementary Fig. 2 Wet and dry weight based concentrations of cadmium in hepatopancreas of shore crabs
(Carcinus maenas) from different sites along the Norwegian coast with different carapace color. Bars denote the mean concentration and whiskers the standard error. The number of crabs within each category is given

Supplementary Fig. 3 Wet and dry weight based concentrations of cadmium in hepatopancreas of shore crabs
(Carcinus maenas) from different sites along the Norwegian coast at different moulting stages. Bars denote the mean concentration and whiskers the standard error. The number of crabs within each category is given

Supplementary Fig. 4 Wet and dry weight based concentrations of Cd in hepatopancreas of male shore crabs
(Carcinus maenas) from different sites along the Norwegian coast at different gonad maturation stages. Bars denote the mean concentration and whiskers the standard error. The number of crabs within each category is given

Supplementary Fig. 5 Wet and dry weight based concentrations of Cd in hepatopancreas of female shore crabs
(Carcinus maenas) from different sites along the Norwegian coast at different gonad maturation stages. Bars denote the mean concentration and whiskers the standard error. The number of crabs within each category is given

Supplementary table 1 Cadmium concentrations (mg/kg wet weight) in muscle meat from claws and female gonads of shore crabs (Carcinus maenas) from the Norwegian coast. Mean ± standard deviation (SD) and concentration ranges are given for each group, except for the measured concentration levels in female gonads, as these results are based on measurements of only one pooled sample (N = 1).

| Area | Tissue | Male | | | Female | | |
| --- | --- | --- | --- | --- | --- | --- | --- |
|  |  | N | Mean ± SD  (mg/kg ww) | Range  (mg/kg ww) | N | Mean ± SD  (mg/kg ww) | Range  (mg/kg ww) |
| Kvitsøy | Muscle | 3 | 0.0053 ± 0.0042 | 0.0028 – 0.010 | 3 | 0.0028 ± 0.00058 | 0.0021 – 0.0032 |
|  | Gonad | - | - | - | 1 | 0.023 | - |
| Sotra-April | Muscle | 3 | 0.0022 ± 0.00063 | 0.0015 – 0.0027 | 3 | 0.0024 ± 0.00033 | 0.0020 – 0.0027 |
|  | Gonad | - | - | - | 1 | 0.017 | - |
| Fleinvær | Muscle | 3 | 0.0027 ± 0.0015 | 0.0017 – 0.0044 | 3 | 0.0037 ± 0.0015 | 0.0020 – 0.0051 |
|  | Gonad | - | - | - | 1 | 0.0092 | - |
| Vesterålen | Muscle | 3 | 0.0018 ± 0.00037 | 0.0015 – 0.0022 | 3 | 0.0020 ± 0.00023 | 0.0018 – 0.0022 |
|  | Gonad | - | - | - | 1 | 0.015 | - |
| Sotra-August | Muscle | 3 | 0.0017 ± 0.0012 | 0.0030 – 0.0012 | 3 | 0.0020 ± 0.0 | 0.0020 – 0.0020 |
|  | Gonad | - | - | - | 1 | 0.015 | - |
| All areas | Muscle | 15 | 0.0027 ± 0.0022 | 0.0010 – 0.010 | 15 | 0.0025 ± 0.00090 | 0.0018 – 0.0051 |
|  | Gonad | - | - | - | 5 | 0.015 ± 0.0055 | 0.0092 – 0.023 |

Supplementary table 2 Cadmium concentrations (mg/kg dry weight) in muscle meat from claws and female gonads of shore crabs (Carcinus maenas) from the Norwegian coast. Mean ± standard deviation (SD) and concentration ranges are given for each group, except for the measured concentration levels in female gonads, as these results are based on measurements of only one pooled sample (N = 1).

| Area | Tissue | Male | | | Female | | |
| --- | --- | --- | --- | --- | --- | --- | --- |
|  |  | N | Mean ± SD  (mg/kg dw) | Range  (mg/kg dw) | N | Mean ± SD  (mg/kg dw) | Range  (mg/kg dw) |
| Kvitsøy | Muscle | 3 | 0.022 ± 0.016 | 0.013 – 0.041 | 3 | 0.012 ± 0.0013 | 0.010 – 0.013 |
|  | Gonad | - | - | - | 1 | 0.050 | - |
| Sotra-April | Muscle | 3 | 0.0087 ± 0.0026 | 0.0059 – 0.011 | 3 | 0.0095 ± 0.0010 | 0.0083 – 0.010 |
|  | Gonad | - | - | - | 1 | 0.044 | - |
| Fleinvær | Muscle | 3 | 0.013 ± 0.0083 | 0.0062 – 0.022 | 3 | 0.015 ± 0.0067 | 0.0080 – 0.021 |
|  | Gonad | - | - | - | 1 | 0.020 | - |
| Vesterålen | Muscle | 3 | 0.0069 ± 0.0015 | 0.0058 – 0.0086 | 3 | 0.0086 ± 0.0010 | 0.0074 – 0.0094 |
|  | Gonad | - | - | - | 1 | 0.022 | - |
| Sotra-August | Muscle | 3 | 0.0072 ± 0.0045 | 0.0044 – 0.012 | 3 | 0.0085 ± 0.00036 | 0.0084 – 0.0085 |
|  | Gonad | - | - | - | 1 | 0.044 | - |
| All areas | Muscle | 15 | 0.012 ± 0.0093 | 0.0044 – 0.041 | 15 | 0.011 ± 0.0036 | 0.0074 – 0.021 |
|  | Gonad | - | - | - | 5 | 0.036 ± 0.014 | 0.020 – 0.050 |

**Supplementary table 3** r- and p-values for significant correlations (Pearson's linear correlation) between cadmium and physiological variables. The significance level was set at 0.05

| Variables | Male shore crabs | Female shore crabs |
| --- | --- | --- |
| Carapace with (cm) and whole wet weight (g) | r = 0.9498***, r^2^ = 0.90 for all locations | r = 0.9404***, r^2^ = 0.88 for all locations |
| Carapace width (cm) and water content in HP (%) | r = 0.4989***, r^2^ = 0.25 for all locations | r = 0,5633*** , r^2^ = 0.32 for all locations |
| Carapace width (cm) and HSI (%) | r = -0.5954*** , r^2^ = -0.35 for all locations | - |
| Carapace width (cm) and Cd (mg/kg ww) | r = 0.2233** , r^2^ = 0.050 for all locations  r = 0.3983**, r^2^ = 0.15 for Vesterålen | r = -0.5448*, r^2^ = -0.29 for Sotra-August |
| Carapace width (cm) and Cd (mg/kg dw) | r = 0.3030***, r^2^ = 0.092 for all locations  r = 0.5344***, r^2^ = 0.29 for Vesterålen | - |
| Carapace width (cm) and Cd content (mg) in HP | r = 0.5312*** , r^2^ = 0.28 for all locations  r = 0.3594*, r^2^ = 0.13 for Sotra-April  r = 0.6872***, r^2^ = 0.47 for Vesterålen | r = 0.2824*, r^2^ = 0.080 for all locations  r = 0.3835*, r^2^ = 0.15 for Sotra-April |
| Water content in HP (%) and Cd (mg/kg ww) | r = 0.1713* r^2^ = 0.029 for all locations  r = 0.4424*, r^2^ = 0.20 for Vesterålen | - |
| Water content in HP (%) and Cd (mg/kg dw) | r = 0.3574*** r^2^ = 0.13 for all locations  r = 0.6260***, r^2^ = 0.39 for Vesterålen | - |
| Water content in HP (%) and Cd content (mg) | r = 0.2074* r^2^ = 0.043 for all locations  r = 0.5493***, r^2^ = 0.30 for Vesterålen | - |
| HSI (%) and Cd (mg/kg ww) | r = -0.3511* , r^2^ = 0.12 for Vesterålen | r = -0.2244* , r^2^ = 0.050 for all locations  r = -0.4529*, r^2^ = -0.21 for Sotra-April |
| HSI (%) and Cd (mg/kg dw) | r = -0.2549*, r^2^ = -0.065 for all locations  r = -0.5163***, r^2^ = 0.27 for Vesterålen | r = -0.2249*, r^2^ = -0.051  r = -0.5078**, r^2^ = -0.25 |
| HSI (%) and Cd (mg) | r = -0.3548*, r^2^ = 0.13 for Vesterålen | - |

**p < 0.05, **p < 0.001, ***p < 0.0001*
